# Fluoxetine treatment is effective in a rat model of childhood-induced post-traumatic stress disorder

**DOI:** 10.1038/s41398-017-0014-5

**Published:** 2017-11-30

**Authors:** Lior Ariel, Sapir Inbar, Schachaf Edut, Gal Richter-Levin

**Affiliations:** 1 Psychology Department, University of Haifa, Haifa, Israel; 2 The Integrated Brain and Behavior Research Center (IBBR), University of Haifa, Haifa, Israel; 3 Sagol Department of Neurobiology, University of Haifa, Haifa, Israel

## Abstract

Although selective serotonin reuptake inhibitors (SSRIs) are first-line treatment for post-traumatic stress disorder (PTSD) patients, their therapeutic efficacy is limited. Childhood adversities are considered a risk factor for developing PTSD in adulthood but may trigger PTSD without additional trauma in some individuals. Nevertheless, just as childhood is considered a vulnerable period it may also be an effective period for preventive treatment. Using a rat model of childhood-induced PTSD, pre-pubertal stress (juvenile stress, JVS), we compared the therapeutic effects of fluoxetine and examined the effectiveness of 1 month of fluoxetine treatment following JVS and into adulthood compared to treatment in adulthood. Since not all individuals develop PTSD following a trauma, comparing only group means is not the adequate type of analysis. We employed a behavioral profiling approach, which analyzes individual differences compared to the normal behavior of a control group. Animals exposed to JVS exhibited a higher proportion of affected animals as measured using the elevated plus maze 8 weeks after JVS. Fluoxetine treatment following the JVS significantly decreased the proportion of affected animals as measured in adulthood. Fluoxetine treatment in adulthood was not effective. The results support the notion that childhood is not only a vulnerable period but also an effective period for preventive treatment.

## Introduction

Post-traumatic stress disorder (PTSD) is highly prevalent in adults that suffered childhood abuse^1,2^. Approximately one in six children and adolescents (16%) develop PTSD after exposure to a DSM-IV criterion A1 or DSM-V trauma. Variation was related to type of trauma and gender, with interpersonal trauma leading to higher rates of PTSD and girls being at higher risk than boys^3^. There is extensive evidence that survivors of childhood abuse tend to show high levels of symptom complexity beyond PTSD, including emotion regulation difficulties, interpersonal problems, impulsive and/or self-destructive behavior, high levels of dissociation, substance-related problems, or somatic symptoms^4,5^. Additionally, children seem to be more sensitive to the effects of trauma, and early life trauma exposure may induce a complex sequence of events that leads to the development of multiple psychiatric disorders in adulthood^6^.

The lasting psychological impact of exposure to trauma in childhood is also accompanied by enduring neurophysiological changes manifested in adulthood. Different studies and meta-analyses repeatedly found structural abnormalities in persons with PTSD compared to controls with and without trauma exposure. These abnormalities are different between adulthood PTSD and pediatric PTSD. The main findings in adulthood are significantly smaller hippocampal, amygdala and anterior cingulate cortex volumes, while pediatric samples exhibit significantly smaller corpus callosum and frontal lobe volumes in PTSD compared to controls^7–11^. It was found that following childhood trauma the urinary concentrations of important neuromodulators such as dopamine, noradrenaline, and cortisol were higher in individuals with PTSD^12^. Childhood trauma was associated with short leukocyte telomere length in adults with chronic PTSD^13^. Childhood maltreatment was also associated with distinct genomic and epigenetic profiles in PTSD, providing a genome-wide evidence of distinct biological modifications in PTSD in the presence or absence of exposure to childhood abuse. Non-overlapping biological pathways seemed to be affected in a PTSD childhood-abused group and a non-childhood-abused PTSD group^14^. These findings in humans may reflect differences in the pathophysiology of PTSD, in dependence of exposure to childhood maltreatment.

Selective serotonin reuptake inhibitors (SSRIs), including fluoxetine, are considered as first-line medication treatments for PTSD. These medications are the most extensively studied and have demonstrated efficacy in reducing core PTSD symptoms, both as short and long-term treatment^15–17^. However, even when treated with these first-line treatment, response rates rarely exceed 60% and less than 20–30% of the patients achieve full remission^18, 19^.

Similar to other psychiatric conditions during childhood, childhood PTSD is treated usually using psychotherapy, and to a lesser extent with pharmacological agents. Thus, there are fewer studies regarding pharmacological treatments in childhood PTSD. Only within the last decade, pharmacological treatments in children have been subjected to randomized clinical trials. In general, the development of these pharmacological interventions has been largely based on data from medication trials in adults with PTSD. Childhood PTSD is highly comorbid with other psychiatric disorders and SSRIs are effective for the treatment of pediatric anxiety disorders^20^ and depression^21^. So far, only a few trials of SSRIs were conducted in youth and they did not suggest a conclusive benefit for PTSD symptoms^22^; one out of three trials found an improvement and two trials did not, but in one of them the pharmacological treatment was adjunctive to a highly effective psychological treatment, which likely made the detection of any potential pharmacological-related improvement difficult. A small body of literature suggests efficacy of several psychopharmacological interventions as monotherapy for pediatric PTSD (antiadrenergic agents like alpha-2 agonizts and alpha-1 antagonists, several second-generation antipsychotics, and several antiepileptic agents)^7^.

In light of the differences between childhood PTSD and PTSD during adulthood, the low response rates to SSRIs in adulthood PTSD, and the urgent need of examining the efficacy of pharmacological treatment of childhood PTSD, we aimed in the current study to compare between the effect of an early pharmacological intervention using fluoxetine during juvenility and the effect of a later intervention, during adulthood. Research indicates that juvenility is a period of great plasticity of the brain^23^. Thus, we assumed that juvenility may be not only a sensitive period for vulnerability but also for intervention. We hypothesized that an early intervention during juvenility may be more effective than a later one during adulthood. We used a juvenile stress procedure (JVS)^24^ to model a childhood adverse experience in rats and examined the behavioral effects of fluoxetine treatment in the two different time points. To better model PTSD we evaluated long-term behavioral reaction to JVS using a behavioral profiling approach^25^ in order to analyze individual differences and to identify only emotionally affected animals among the entire stress-exposed population. Thus, instead of inferring only from significant differences in the average score of the behavior assessed, we were able to examine the relative proportion of affected vs. non-affected individuals and thus better mimic the clinical assessment of the pathology^25–28^.

## Materials and methods

All experimental procedures and assessments were conducted in accordance with the NIH guidelines for the care and use of laboratory animals and were approved by the University of Haifa ethical committee (Ethical Nr. 342/14). One-hundred sixteen adult male Sprague Dawley rats, post-natal day (PND) 22, weighing 35–49 g (Harlan, Jerusalem, Israel) at arrival, were habituated in the laboratory vivarium for 5 days. Animals were housed 3–4 per cage in a temperature controlled (22 ± 2 °C) animal quarters on a 12:12-h light–dark cycle (lights on at 7:00 a.m.–7:00 p.m.). They had ad libitum access to standard rodent chow pellets and water. Our group size meets at least a minimum number necessary to address the experimental objectives in a meaningful fashion. This is based largely upon personal experience as well as the group sizes typically appearing in the literature.

### The juvenile stress protocol (JVS)

This protocol (adapted from Tsoory et al.^24^) is a 3-day exposure to different stressors (detailed below) applied during juvenility (PNDs 27–29) and serves as an animal model for childhood adversity^29^. All procedures were conducted under full light illumination.

- Day 1 (PND 27) Forced swim. 10 min forced swim in an opaque circular water tank (diameter 0.5 m; height 0.5 m; water depth 0.4 m), water temperature 22 ± 2 °C.
- Day 2 (PND 28) Elevated platform. Three 30 min trials; Inter-Trials Interval (ITI): 60 min in the home cage. Elevated platform: A Perspex surface covered with a black coarse rubber (12 × 12 cm) 70 cm above floor level, located in the middle of a small closet-like room.
- Day 3 (PND 29) Restrain. Rats were placed in a metal mesh restraining box (11 × 5 × 4 cm) that prevents forward-backward movement and limits side-to-side mobility. Rats remained in the restraining box for 2 h.

### Fluoxetine treatment through drinking water (FLX)

Adapted from McNamara et al.^30^ 24 h water consumption was measured for each cage using bottle weights (1 g water = 1 ml water) for 3 days prior to drug delivery. FLX daily dosage of 10 mg/kg/day was diluted in drinking water for the FLX-treated groups. This dosage was selected based on studies demonstrating that it produces clinically-relevant plasma concentrations, reduces cortical serotonin turnover in rats, and reduces behavioral indices of depression in the forced swim test^31, 32^. Fresh solutions were prepared twice a week using FLX stock solution (3 mg/ml) (Vetmarket, Petah-Tikva, Israel) that was added to drinking water at the required concentration. FLX concentration was determined according to average daily fluid consumption and body weight that were measured twice a week and once a week, respectively. Amber opaque drinking bottles were used to protect FLX from light degradation. All other rats were receiving regular drinking water.

### The elevated plus maze test (EPM)

Adapted from Pellow et al.^33^ Following 2 min habituation to the testing room rats were placed on the central-platform of the maze facing an open arm (110 × 110 cm, 70 cm above the floor; two opposing open arms/closed arms surrounded by 40 cm high opaque walls on three sides; the maze is covered with a black plexiglas; full light illumination), and were allowed to explore the maze for 5 min. Rats’ behavior was tracked, recorded, and analyzed by the EthoVision XT8 tracking system (Noldus Information Technology, Wageningen, The Netherlands). Behavioral measures included distance covered, duration of stay, and number of entrances in the different arms of the maze.

### Experimental design

**Fig. 1:**
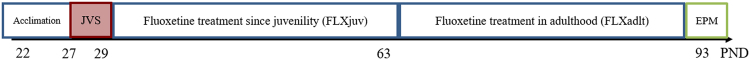
Timeline of the experimental design

The animals were randomly assigned to the different experimental groups. No blinding was used. Following habituation in the vivarium, rats were either exposed to JVS (PND 27–29 s) or not. All rats were either exposed to regular drinking water throughout the experiment, or chronically treated with FLX via drinking water for 30–33 days following JVS procedure (FLXjuv, PND 30–63), or during adulthood (FLXadlt, PND 64–93). At PND 93, all rats were assessed using the EPM test. The timeline of our design is summarized in Fig. 1.

### Behavioral profiling

**Fig. 2:**
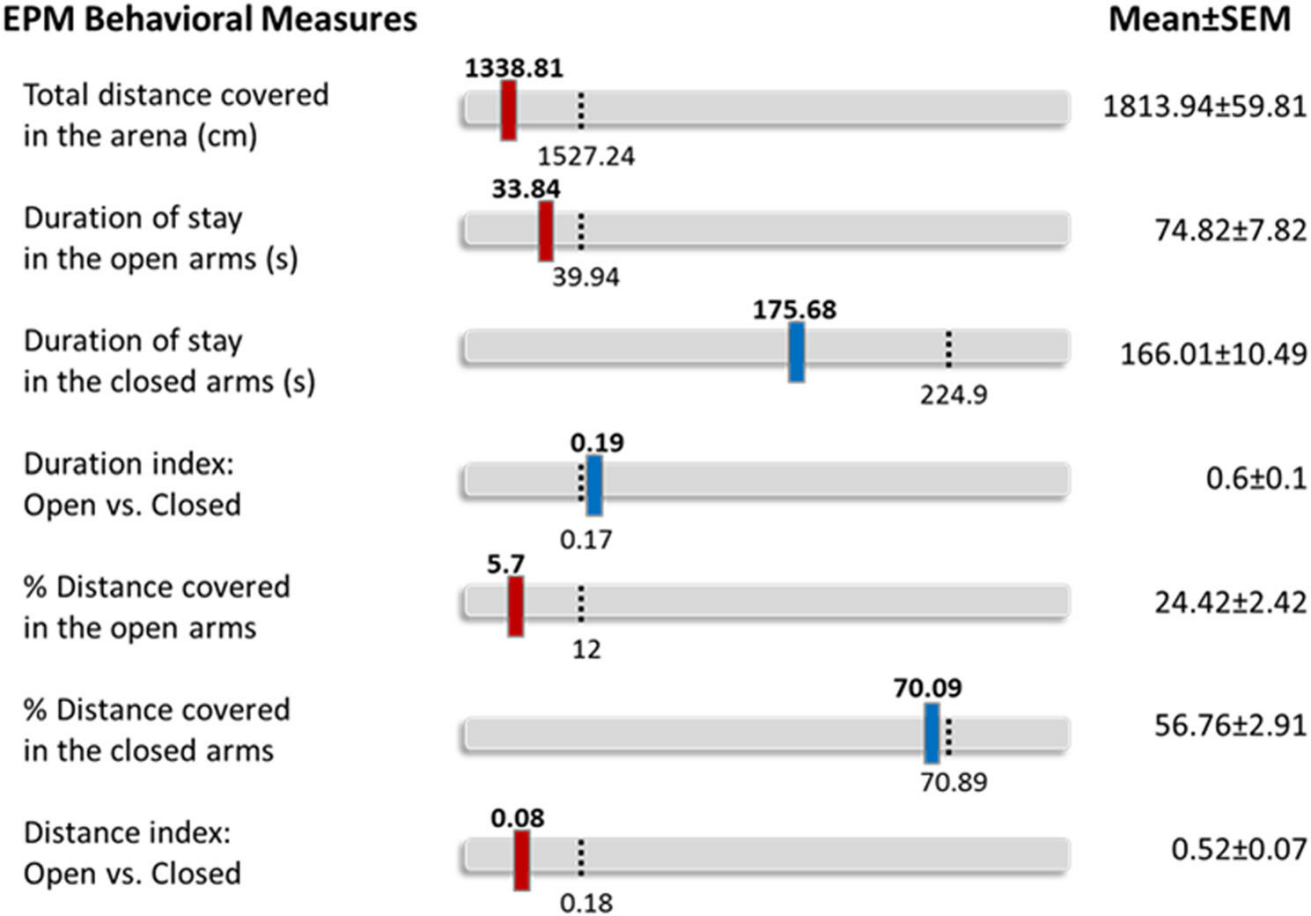
A representative figure of the performances of an “affected” animal Numbers in bold represent this animal’s particular scores. Blue bars represent the animal’s scores that are within normal distribution, while the red bars represent the animal’s scores that deviate from normal distribution. Since 4 out of 7 measures deviate from the normal distribution, this animal was classified as “affected”

Profiling animals as “affected” or “unaffected” was based on the cutoff behavioral criteria (CBC) analysis approach originally proposed by Cohen et al.^34^ and further developed by Ardi et al.^25^. We employed a behavioral profiling approach, which analyzes individual differences compared to the norm. In order to create a behavioral classification, we referred to the performance of the control group (*n* = 30) as the behavior of the normal population. We first determined the distribution of measures in the control group and calculated the lower 20th percentages (or upper depending on the measure). This approach was applied on seven different parameters of distance and duration in the EPM, which represent anxiety-like behaviors. The measures of each animal were compared to the distribution curve of the control group. In order to be classified as affected, an animal must exhibit values that are under/above the 20th percentages in at least 4 of the 7 measures. Figure 2 shows the representative performances of an “affected” animal.

### Statistical analysis

Weight of animals was analyzed using repeated measures two-way analysis of variance (ANOVA) with the stress (control, JVS) and the drug treatment (no FLX, FLXjuv, FLXadlt) as factors. Fluid intake was analyzed separately for the two treatment periods using repeated measures two-way ANOVA with the stress (control, JVS) and the drug treatment (water, FLX) as factors.

Behavioral differences in the EPM were first analyzed by using two-way ANOVA with the stress (control, JVS) and the drug treatment (no FLX, FLXjuv, FLXadlt) as factors, followed by Bonferroni post hoc tests for the drug treatment factor when needed. Then, individual behavioral profiling was applied and the distribution of affected vs. unaffected populations was calculated by using Chi-squared test for goodness of fit.

All tests were selected after confirming data meet the assumptions of the tests. Data were analyzed using the IBM SPSS Statistics Software version 19 (IBM, Armonk, NY, USA).

## Results

### Weight and fluid intake

Repeated measures two-way ANOVA revealed no significant differences in the animals’ weight between groups [stress: *F*_(1,98)_ = 0.09, NS; drug: *F*_(2,98)_ = 0.46, NS; stress X drug: *F*_(2,98)_ = 1.07, NS].

**Fig. 3:**
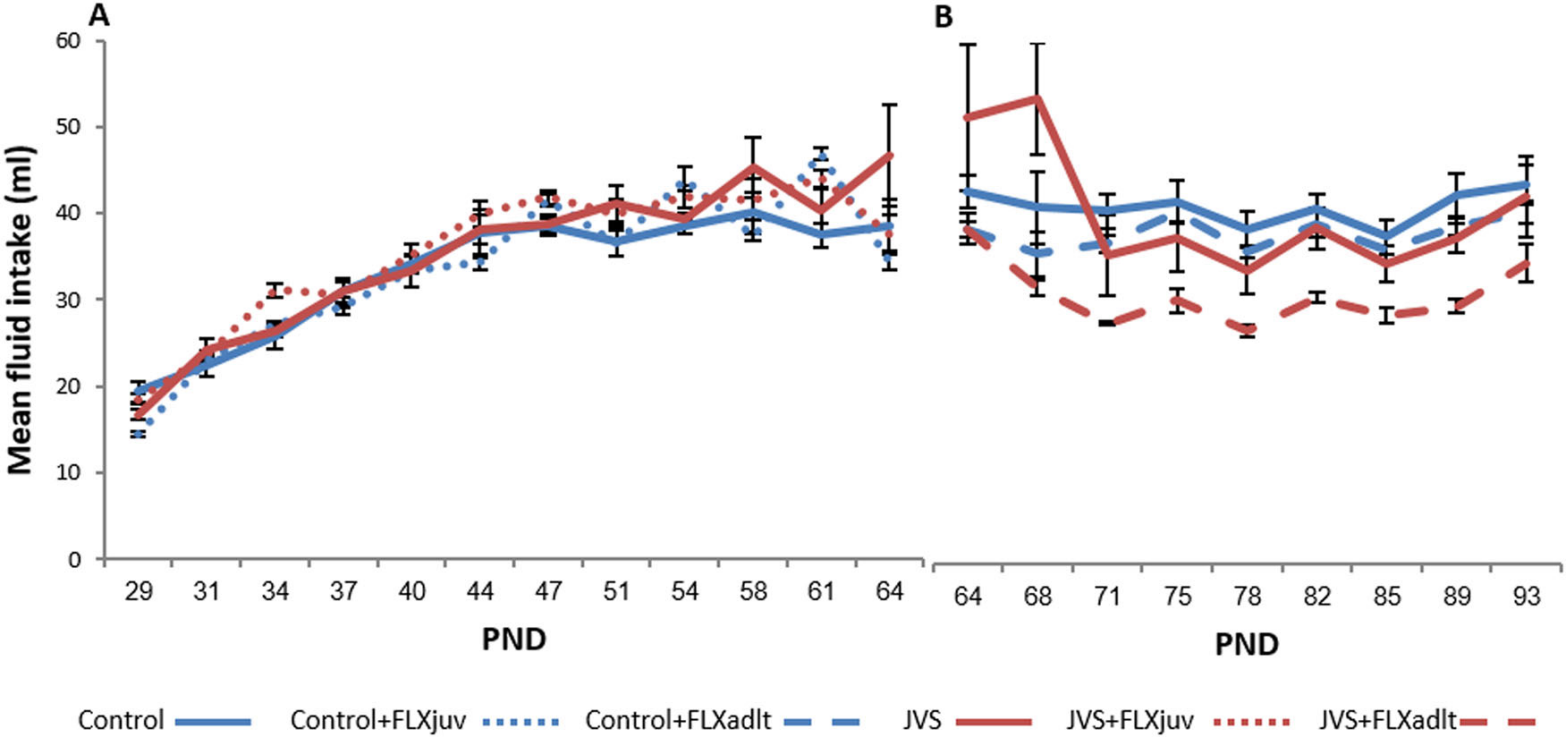
Effects of juvenile stress (JVS) and fluoxetine treatment (FLX) on fluid intake Fluid intake measured when FLX was given since juvenility (**a**). Fluid intake measured when FLX was given during adulthood (**b**). All rats that were treated with FLX had decreased fluid intake compared to all rats that were drinking water, but only when treated during adulthood (*p* < 0.01). Data expressed in means ± SEM. N since juvenility: control = 10, control + FLXjuv = 9, JVS = 9, JVS + FLXjuv = 16; N during adulthood: control = 17, control + FLXadlt = 12, JVS = 6, JVS + FLXadlt = 12

Repeated measures two-way ANOVA for fluid intake during the FLX treatment revealed a significant effect for the drug treatment only during adulthood, indicating that rats that were treated with FLX had decreased fluid intake compared to animals that were drinking water only when treated during adulthood (Fig. 3). No other effects were found [since juvenility - stress: *F*_(1,40)_ = 3.12, NS; drug: *F*_(1,40)_ = 0.07, NS; stress X drug: *F*_(1,40)_ = 0.01, NS; during adulthood - stress: *F*_(1,43)_ = 3.64, NS; drug: *F*_(1,43)_ = 10.28, *p* < 0.01; stress X drug: *F*_(1,43)_ = 2.74, NS].

### Averaged group effects in the Elevated plus maze

**Fig. 4:**
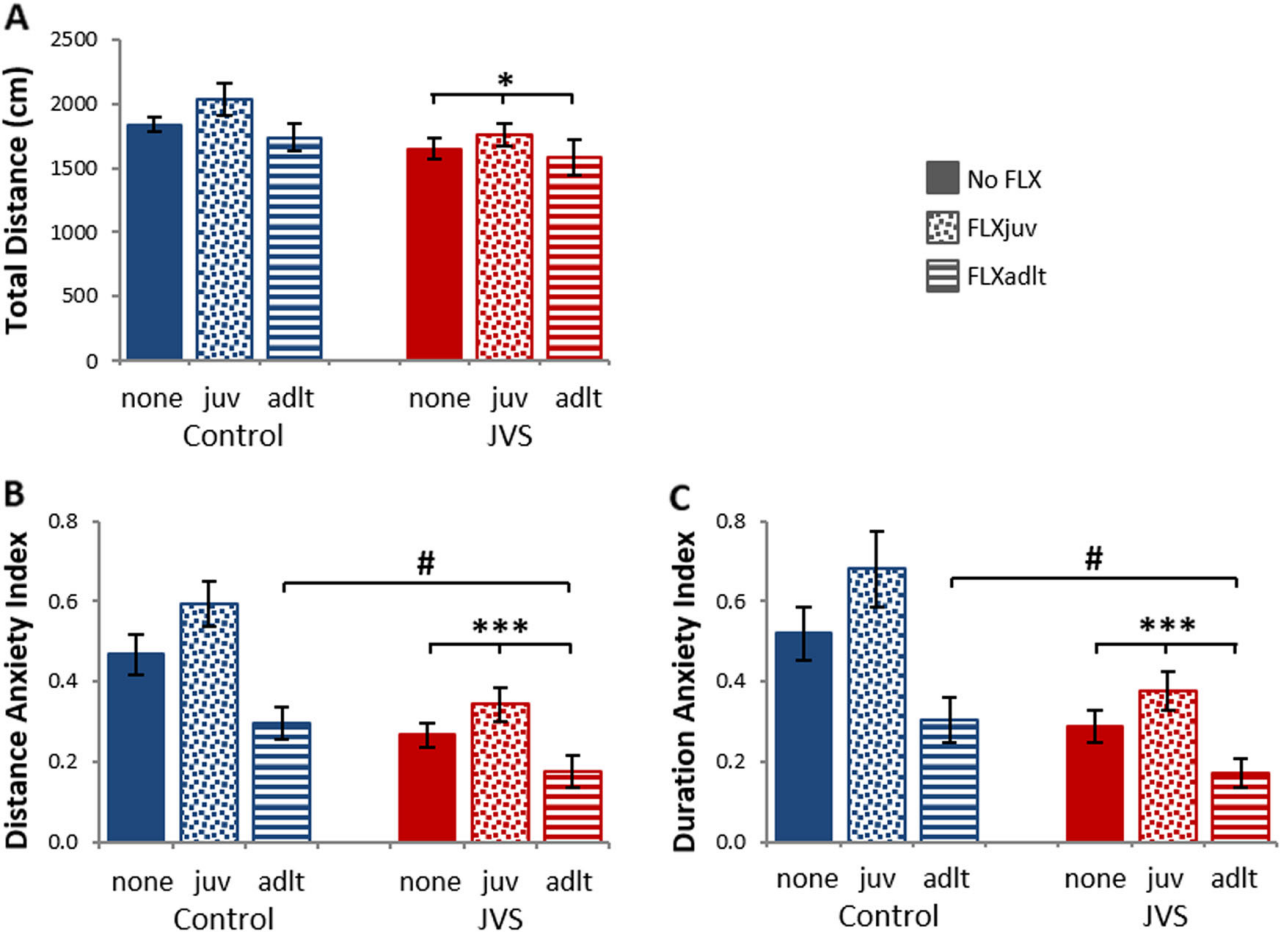
Effects of juvenile stress (JVS) and fluoxetine treatment (FLX) on anxiety-like behavior in the elevated plus maze Total distance covered in the maze (**a**). Distance anxiety index measured as open / closed arms ratio, with lower ratios indicating higher anxiety levels (**b**). Duration anxiety index measured as open /closed arms ratio, with lower ratios indicating higher anxiety levels (**c**). All JVS groups exhibited significantly lower activity level and significantly higher levels of anxiety by both distance and duration indexes, compared to control rats. FLXadlt groups exhibited significantly higher levels of anxiety by both distance and duration indexes, compared to “no FLX” groups, while FLXjuv groups were not significantly different from “no FLX” groups. Data expressed in means ± SEM. N: control = 30, control + FLXjuv = 12, control + FLXadlt = 12, JVS = 31, JVS + FLXjuv = 19, JVS + FLXadlt = 12. *Significant difference compared to control groups, *p* < 0.05, ***p < 0.001. ^#^Significant difference compared to no FLX groups, *p* < 0.05

Two-way ANOVA revealed a significant difference between JVS animals and controls in general activity level as was measured by total distance covered in the maze (Fig. 4a) [*F*_(1,110)_ = 6.38, *p* < 0.05], as well as in distance and duration anxiety indexes (Fig. 4b, c) measured as open / closed arms ratio, with lower ratios indicating higher anxiety levels [*F*_(1,110)_ = 20.79, *p* < 0.001; *F*_(1,110)_ = 17.22, *p* < 0.001; respectively]. Rats that were exposed to JVS exhibited significantly lower activity level and significantly higher levels of anxiety by both distance and duration indexes, compared to control rats.

The analysis also revealed a significant main effect for the drug treatment in distance and duration anxiety indexes but not in the total distance [*F*_(2,110)_ = 8.41, *p* < 0.001; *F*_(2,110)_ = 7.92, *p* < 0.001; *F*_(2,110)_ = 2.47, NS; respectively]. Post hoc tests showed that animals that were treated with FLX during adulthood exhibited significantly higher levels of anxiety by both distance and duration indexes, compared to all other rats (*p* < 0.05). However, rats that were treated with FLX since juvenility were not significantly different from rats that did not receive FLX. They demonstrated lower levels of anxiety by both distance and duration indexes, though this trend was non-significant.

The interaction was not significant in all measures [Total distance: *F*_(2,110)_ = 0.19, NS; distance anxiety index: *F*_(2,110)_ = 0.69, NS; duration anxiety index: *F*_(2,110)_ = 0.7, NS].

### Behavioral profiling and individual differences analysis

**Fig. 5:**
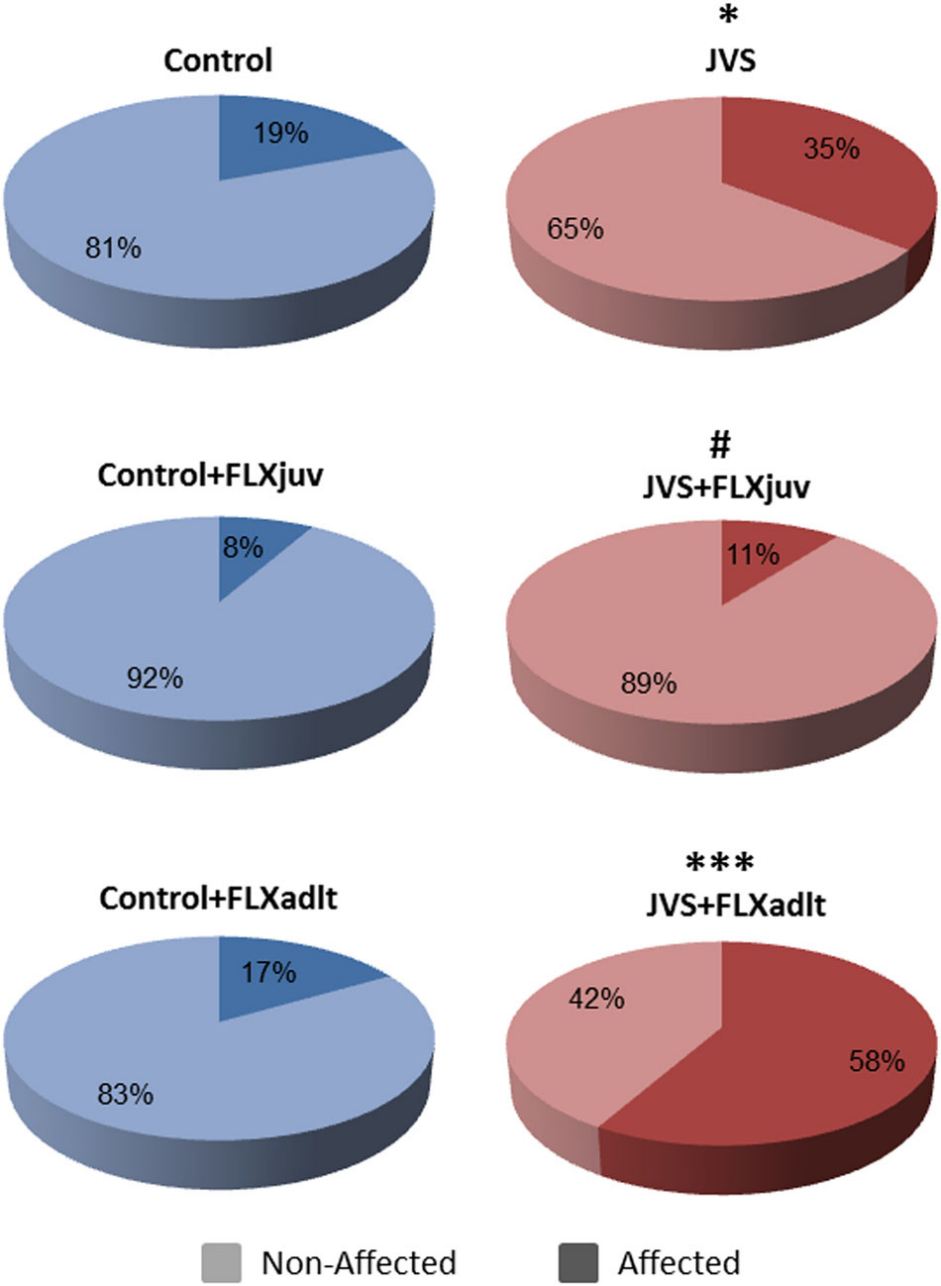
The effects of juvenile stress (JVS) and fluoxetine treatment (FLX) on the distribution of affected and non-affected animals JVS Rats exhibited significantly higher rates of affected animals compared to controls. Following JVS, only FLXjuv rats exhibited significantly lower rates of affected animals compared to JVS, rates that are similar to the control group. N: control = 30, control + FLXjuv = 12, control + FLXadlt = 12, JVS = 31, JVS + FLXjuv = 19, JVS + FLXadlt = 12. *Significant difference compared to controls, *p* < 0.05, ***p < 0.001, ^#^Significant difference compared to JVS group, *p* < 0.05

Following behavioral profiling, Chi-squared test for goodness of fit was used in order to examine individual differences (Fig. 5). The distribution between affected and non-affected animals in each group was compared to the expected distribution in the control group (20:80). Chi-squared test for goodness of fit to a distribution ratio of 20:80 for affected: non-affected animals, revealed no significant difference for the distributions of the different control groups [non-treated control: *χ* ^2^_(1)_ = 0, n.s.; control + FLXjuv: *χ* ^2^_(1)_ = 1.02, n.s.; control + FLXadlt: *χ* ^2^_(1)_ = 0.08, n.s.]. The test did reveal a significant difference for the distributions of JVS [*χ* ^2^_(1)_ = 4.65, *p* < 0.05], and JVS + FLXadlt [*χ* ^2^_(1)_ = 11.02, *p* < 0.001]. However, there was no significant difference for the distribution of JVS + FLXjuv [*χ* ^2^_(1)_ = 1.07, n.s.]. Rats that were exposed to JVS exhibited significantly higher rates of affected animals. Only when rats were exposed to JVS and treated with fluoxetine since juvenility their proportion of affected animals was similar to controls.

Within animals that were exposed to JVS, Chi-squared test for goodness of fit to the distribution ratio of non-treated JVS rats (35:65) for affected: non-affected animals, revealed a significant difference only for the distribution of rats that were treated with FLX since juvenility and not for the rats that were treated during adulthood [JVS + FLXjuv: *χ* ^2^_(1)_ = 5, *p* < 0.05; JVS + FLXadlt: *χ* ^2^_(1)_ = 2.87, n.s.]. Following JVS, only rats that were treated with FLX since juvenility exhibited significantly lower rates of affected animals, rates that are similar to the control group.

## Discussion

We found that the exposure to JVS induced a lasting increase in anxiety-like behavior in the EPM, as well as an enlarged proportion of animals characterized as emotionally affected. This finding replicates different studies indicating that JVS results in long-term behavioral impairments that were associated with various neurobiological alterations^29^. It is highly possible that the stress in juvenility has served as the “trauma” that induced anxiety-like symptoms lasting throughout adulthood. These results suggest once again that juvenility is a sensitive period for vulnerability.

Analyzing the averaged data of groups FLX treatment seems to have a somewhat surprising anxiogenic effect regardless of exposure to JVS. While there have been some reports of an anxiogenic effect of FLX^21, 35^ these are the exception. However, as was discussed in detail in Ardi et al.^25^, using averaged data when trying to model PTSD may be problematic. Most individuals who were exposed to trauma do not develop PTSD and do not exhibit symptoms in the long-run. Looking at the average score masks individual differences between animals. In contrast, employing the behavioral profiling approach, assessing differences in the proportions of affected animals in each group is preferred when modeling PTSD. Indeed, employing the behavioral profiling approach it was revealed that FLX intervention in juvenility significantly lowered the proportion of affected animals that were exposed to JVS and brought it to be similar to that of the control group. However, the later FLX intervention, during adulthood, did not lower the proportion of affected animals.

Our finding that on the average, FLX given during adulthood significantly increases anxiety-like behavior as measured in the EPM may be result from individual differences in responding to FLX. Another possible reason could be the timing of the test. The animals that were treated with fluoxetine during adulthood received the FLX until a day before the EPM test, while the animals that were treated since juvenility were tested a month after the treatment. This may be one reason for this negative effect of fluoxetine given during adulthood. Nevertheless, the finding lands further support to the possibility that given since juvenility fluoxetine may have less side effects than when is given during adulthood.

Our results indicate that the juvenility period is not only a sensitive period for vulnerability as was argued above; it may also be a sensitive period in a positive way and may serve as a critical time point for intervention. Similar results were found in studies where enriched environment (EE) was used as an early intervention following JVS. In one study EE that was given following JVS and until adulthood could reverse most of the effects of JVS, both at the behavioral, endocrine, and biochemical levels^36^. In another study, it was found that an exposure to EE in adulthood was ineffective in preventing the behavioral effects of an exposure to the combination of JVS and another stress exposure during adulthood, while EE in juvenility could prevent these effects. In addition, while protein expression levels of the GABAA receptor subunit α1 in the dorsal dentate gyrus of the hippocampus remained high, an exposure to EE in juvenility could restore the protein expression levels back to control levels in both basolateral amygdala and the ventral dentate gyrus (Ardi, Richter-Levin & Richter-Levin, unpublished). Apparently environmental treatment as well as pharmacological treatment during juvenility has a beneficial effect over treatment given only during adulthood.

In light of these results it is reasonable to expect that in humans, treatment during juvenility will also have better results, compared to treatment later on in life, only during adulthood. As similar to other psychiatric conditions during childhood, PTSD is treated usually using psychotherapy, and to a lesser extent with pharmacological agents. Thus, there are fewer studies regarding pharmacological treatments in children PTSD. Strong evidence supports the efficacy of trauma-focused psychotherapies for the treatment of childhood PTSD^22^. Studies of psychotherapies for traumatized children (including trauma-focused cognitive-behavioral therapy and child–parent psychotherapy) have indicated that they can positively impact a broad range of outcome variables (affective dysregulation, behavioral problems, shame and guilt-related cognitions, and interpersonal functioning) as well as improving symptoms of PTSD^37, 38^. Further, there is some evidence that family-based interventions targeting behavioral change in children can alter hypothalamic–pituitary–adrenal (HPA) axis functioning^39, 40^, and a recent study linked the changes in HPA reactivity to treatment-induced changes in behavior^41^. It is possible, therefore, that early intervention in a manner that addresses the developmental, behavioral, and emotional sequelae of childhood adversities could potentially alter a trajectory to adult PTSD by reducing the impact of later exposure to stressors, also in part by regulating HPA axis stress responsivity that may have been negatively affected by the childhood trauma^42^. This early intervention may be a pharmacological treatment as a stand-alone or as an add-on treatment combined with psychotherapy.

In this article, we focused on PTSD but child adversity may also predispose to other anxiety and mood disorders. SSRIs are used often in these disorders but with varying levels of success. Thus, the current findings may have wider-ranging implications for many disorders associated with childhood adversities, in addition to its relevance to childhood-induced PTSD.

As mentioned above only the minority of children and adolescents develop PTSD after exposure to a trauma. Also in our rat model only 35% of the JVS animals were found affected in adulthood. In the current study, we treated all animals that were exposed to JVS and the diagnostic behavioral test was conducted only during adulthood. Future studies should try to identify predictive behavioral measures that would enable identifying those individuals with higher risk for being affected, in order to focus the treatment only on those. To this date there is no research in humans, examining the relationship between pharmacological treatment of childhood PTSD and the resultant long-term physical and mental health. Also, no study has compared systematically between the effect of pharmacological treatment during childhood and the effect of treatment during adulthood on PTSD that was resulted during childhood. Only few studies are reported, studying the effects of SSRIs on childhood PTSD, with non-conclusive findings. Using our animal model, we found that FLX treatment during juvenility was beneficial compared to that of a later intervention during adulthood. Thus, we suggest that juvenility is a sensitive period for vulnerability, but also for intervention. Juvenility may serve as a critical period for pharmacological as well as psychological intervention, but future studies in humans are needed to verify this notion.
